# The Nigerian Emergency Care System: Structural Challenges, Workforce Constraints, and Financing Gaps

**DOI:** 10.7759/cureus.115854

**Published:** 2026-09-06

**Authors:** Marcel F Jumbo, Boma Oyan, Emmanuel O Nyeche

**Affiliations:** 1 Internal Medicine, Rivers State University Teaching Hospital, Port Harcourt, NGA

**Keywords:** emergency care systems, emergency medical services, health system governance, health workforce, nigeria, prehospital care, public health policy, universal health coverage

## Abstract

Emergency Medical Services (EMS) are a critical component of resilient health systems, enabling timely response to trauma, acute medical conditions, and obstetric emergencies. Despite Nigeria’s substantial burden of preventable emergency-related morbidity and mortality, the development of a coherent and fully functional EMS system remains incomplete. Existing initiatives, including the National Emergency Medical Service and Ambulance System (NEMSAS), have demonstrated policy intent but continue to face persistent structural constraints. This narrative review synthesised peer-reviewed literature, national policy documents, and international agency reports to examine governance structures, workforce capacity, and financing mechanisms within Nigeria’s emergency care system. Comparative analysis with selected high- and middle-income country EMS models was undertaken to identify contextually relevant system-level lessons. Findings indicate that EMS limitations in Nigeria reflect interdependent structural weaknesses rather than isolated operational failures. Governance fragmentation within a federal health system has hindered standardisation, coordination, and accountability, while workforce shortages, exacerbated by limited professionalisation and high rates of migration, restrict both prehospital capacity and clinical leadership. Financing remains inconsistent and heavily reliant on public budget allocations and out-of-pocket payments, limiting sustainability and equitable access. These domains interact in a reinforcing cycle that constrains system performance and the implementation of national reforms. Strengthening EMS in Nigeria will therefore require coordinated cross-domain reform that aligns governance authority, workforce professionalization, and sustainable financing within a unified national framework. Recognising emergency care as a public good and prioritising effective policy implementation are essential to improving health system resilience and advancing progress toward universal health coverage.

## Introduction and background

Emergency conditions, including trauma, acute cardiovascular events, obstetric complications, and severe infections, account for a substantial proportion of preventable morbidity and mortality globally [1,2]. Timely access to organised emergency care has been shown to significantly reduce deaths and disability associated with these time-sensitive conditions [1,3]. As a result, Emergency Medical Services (EMS) and broader emergency care systems are increasingly recognised as essential components of effective, resilient health systems and a prerequisite for achieving universal health coverage (UHC) [1,4].

In low- and middle-income countries (LMICs), however, emergency care systems often remain underdeveloped, fragmented, and inadequately resourced [1,5]. Weak pre-hospital care, delayed access to definitive treatment, shortages of trained personnel, and limited system coordination contribute to poor outcomes for acute illness and injury [2,5]. Recognising these challenges, the World Health Organization (WHO) has emphasised the development of integrated emergency care systems as a critical pathway toward UHC and health system strengthening [1,4].

Nigeria, Africa’s most populous country, bears a high burden of emergency conditions arising from road traffic injuries, noncommunicable diseases, maternal emergencies, and infectious diseases [6-8]. Despite this burden, the organisation and delivery of emergency care in Nigeria remain uneven and largely dependent on state-level initiatives, private providers, and informal transport systems [9,10]. While recent policy efforts, including the establishment of the National Emergency Medical Service and Ambulance System (NEMSAS), represent important steps toward strengthening emergency care delivery, their impact has been constrained by persistent system-level challenges [11].

A comprehensive understanding of these challenges is essential for informing policy implementation, guiding investment, and supporting sustainable reform. However, existing literature on emergency care in Nigeria remains fragmented, with many studies focusing on isolated components such as pre-hospital transport, hospital emergency departments, or workforce shortages, rather than examining the emergency care system as an integrated whole [9,12].

Emergency care systems encompass the full continuum of care for acute illness and injury, from the scene of an emergency through pre-hospital response, transport, and facility-based emergency treatment [1,4]. Effective systems are characterised by coordinated governance structures, sustainable financing mechanisms, a trained and retained workforce, and clearly defined pathways linking communities, emergency responders, and healthcare facilities [1].

In high-income countries, structured EMS systems supported by unified emergency access numbers, regulated ambulance services, and specialist-led emergency departments have been associated with improved response times, better clinical outcomes, and greater equity in access to care [13-15]. By contrast, many LMICs, including Nigeria, continue to rely heavily on informal emergency response mechanisms, with limited integration between pre-hospital and hospital-based services [1,9].

Nigeria’s emergency care landscape is shaped by a complex federal health system in which responsibility for service delivery is shared across federal, state, and local governments [10,15]. While this structure allows for decentralised service delivery, it has also contributed to marked variability in EMS availability, quality, and coverage across regions [9,11]. In some urban centres, relatively well-resourced ambulance services and emergency departments exist. In contrast, in many rural and peri-urban areas, emergency care remains largely inaccessible or dependent on private and informal transport [9,11].

Workforce challenges further complicate emergency care delivery in Nigeria. Shortages of trained emergency medical technicians, paramedics, and emergency medicine physicians limit the capacity of both pre-hospital and facility-based services [12,16]. These shortages are exacerbated by limited training opportunities, weak professional recognition, and ongoing migration of skilled healthcare workers to higher-income settings [17,18]. At the same time, financing for emergency care remains inconsistent, with heavy reliance on government budget allocations, donor funding, and out-of-pocket payments, undermining system sustainability and equitable access [11,19].

Recent national policy initiatives, including broader health sector reforms and the operationalisation of NEMSAS, signal growing recognition of the importance of emergency care within Nigeria’s health system [11,15]. Nevertheless, gaps in governance, financing, workforce development, and system integration continue to limit the effectiveness of these efforts. A system-level synthesis of the existing evidence is therefore required to identify key constraints, highlight areas of progress, and inform context-appropriate strategies for strengthening emergency care delivery in Nigeria.

Aim of the review

This narrative review critically examines the structure and performance of Nigeria’s emergency care system, with a particular focus on governance and policy frameworks, workforce capacity, and financing mechanisms. By drawing on published literature and international comparative models, this review seeks to identify key system-level challenges and highlight opportunities for strengthening EMS and emergency care delivery in Nigeria.

## Review

Governance and policy gaps in Nigeria’s Emergency Medical Services (EMS)

Structural Weaknesses in EMS Governance

Effective EMS systems depend on coherent governance structures that define leadership authority, establish accountability, define coordination mechanisms, and enforce standards across the continuum of emergency care [20-22]. In Nigeria, weaknesses in governance are among the most significant barriers to the development of a functional and equitable EMS system. Although multiple policy instruments exist, including the Emergency Treatment and Care Act and broader national health sector reforms, these policies have not translated into a unified, fully operational governance framework with clearly delineated responsibilities across federal, state, and local levels of government [15,23].

Nigeria’s federal structure places substantial responsibility for health service delivery at the state level, yet emergency care requires a high degree of central coordination to ensure uniform standards, interoperability, and rapid response across jurisdictions [1,24]. In practice, this mismatch has resulted in fragmented authority, overlapping mandates, and inconsistent implementation of emergency care policies [9,11]. While some states have developed relatively functional ambulance services and emergency response mechanisms, often driven by political will or donor support, others lack even basic prehospital infrastructure [10,11]. The absence of binding national standards and enforceable regulatory mechanisms allows these disparities to persist, undermining equity in access to emergency care [1,25].

Institutional capacity constraints further weaken governance effectiveness. Regulatory bodies responsible for oversight of ambulance services, emergency facilities, and prehospital providers often lack the technical expertise, financial resources, and legal authority required to enforce standards consistently [21,26]. As a result, emergency care delivery in many settings remains reactive rather than systematised, characterised by limited performance monitoring, weak data systems, and minimal accountability for service failures [23]. This persistent gap between policy formulation and implementation contributes to avoidable delays, poor coordination, and preventable emergency-related mortality [1,5].

Comparative experience underscores the importance of governance coherence in EMS development. In the United Kingdom, emergency care delivery is centrally coordinated within the National Health Service, with national performance standards implemented through regional ambulance trusts and subject to routine performance monitoring [13,27]. In the United States, although EMS delivery is decentralised, federal oversight, national accreditation standards, and data-driven quality assurance mechanisms ensure minimum service consistency across jurisdictions [14,28]. In contrast, Nigeria’s fragmented governance environment limits coordination, weakens oversight, and impedes the development of a resilient national emergency response system [9,11].

Strengthening EMS governance in Nigeria will therefore require more than the existence of policy documents. It demands enforceable regulatory authority, clearly defined intergovernmental roles, robust monitoring and evaluation systems, and sustained political commitment to emergency care as a core public service [1,21]. Without these elements, even well-designed policies are unlikely to translate into measurable improvements in emergency care outcomes. These governance gaps can be conceptualised as a sequential policy development and implementation pathway, in which the absence of standardised formats, defined drafting processes, formal vetting timelines, and routine review mechanisms necessitates strategic revision at multiple stages (Figure 1).

**Figure 1:**
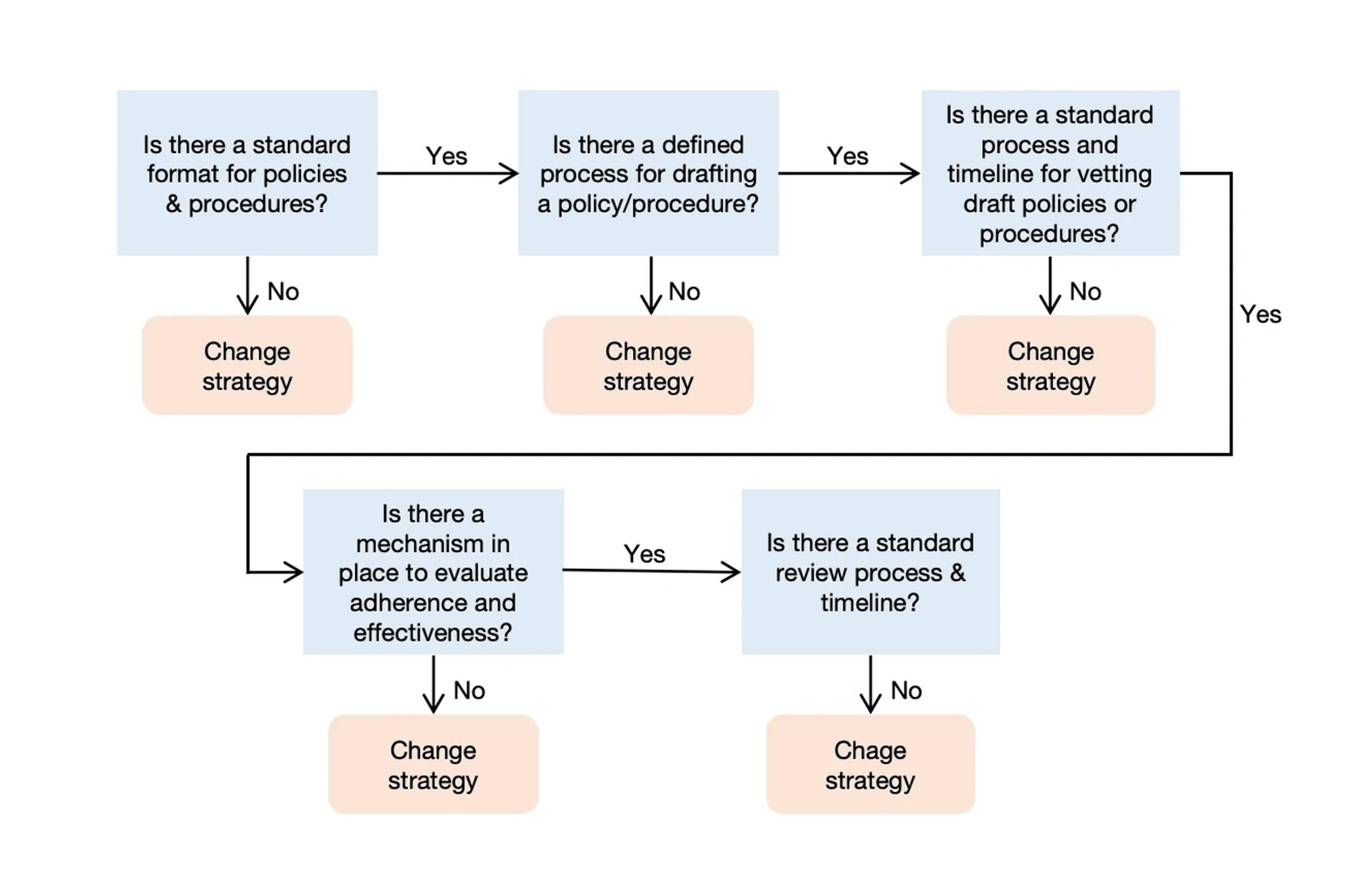
Policy and governance decision pathway for strengthening EMS implementation EMS: Emergency Medical Services This figure illustrates a structured decision-making pathway for evaluating the adequacy of policy and governance processes relevant to the implementation of EMS. Sequential decision points assess the presence of standardised policy formats, defined drafting processes, formal vetting timelines, mechanisms for monitoring adherence and effectiveness, and routine review procedures. At each stage, the absence of a defined standard or process necessitates strategic revision. The framework highlights how gaps in policy development, implementation, and review can undermine system performance, underscoring the importance of coherent governance structures for effective and sustainable EMS delivery. Image credit: Image created by the authors

Absence of a Unified National EMS Framework

The lack of a unified national EMS framework represents a major operational barrier to timely and equitable emergency care in Nigeria, particularly in rural and underserved regions [1,9]. Although the NEMSAS was established to address coordination gaps and standardise EMS delivery, its implementation remains uneven and incomplete across states, limiting its system-wide effectiveness [11,24].

Nigeria currently lacks a nationally recognised single emergency access number. Instead, emergency response relies on a fragmented mix of state-specific numbers, private ambulance hotlines, and informal community networks [9,10]. This fragmentation creates public confusion, delays emergency activation, and reduces the likelihood of timely intervention during critical events [1,25]. In contrast, countries such as the United Kingdom and the United States operate centralised emergency call systems (999 and 911, respectively), enabling rapid dispatch, coordinated triage, and seamless linkage between prehospital and hospital-based care [13,14].

In addition, there is no uniform ambulance classification and regulation system in Nigeria. Ambulances are not consistently categorised according to life-support capabilities, staffing requirements, or equipment standards, resulting in wide variation in the quality of prehospital care delivered [21,26]. Many vehicles used for patient transport lack essential resuscitation equipment and trained personnel, limiting their ability to deliver meaningful prehospital interventions for time-sensitive conditions such as trauma, myocardial infarction, stroke, and obstetric emergencies [1,9]. Weak regulatory oversight of private EMS providers further exacerbates disparities in service quality, affordability, and access [10,25].

Figure 2 illustrates how governance fragmentation, lack of standardised protocols, and weak regulatory oversight collectively hinder the establishment of a unified EMS framework. Addressing these gaps will be critical to improving coordination, response times, and public trust in emergency services, and to ensuring that national EMS reforms translate into equitable population-level benefits.

**Figure 2:**
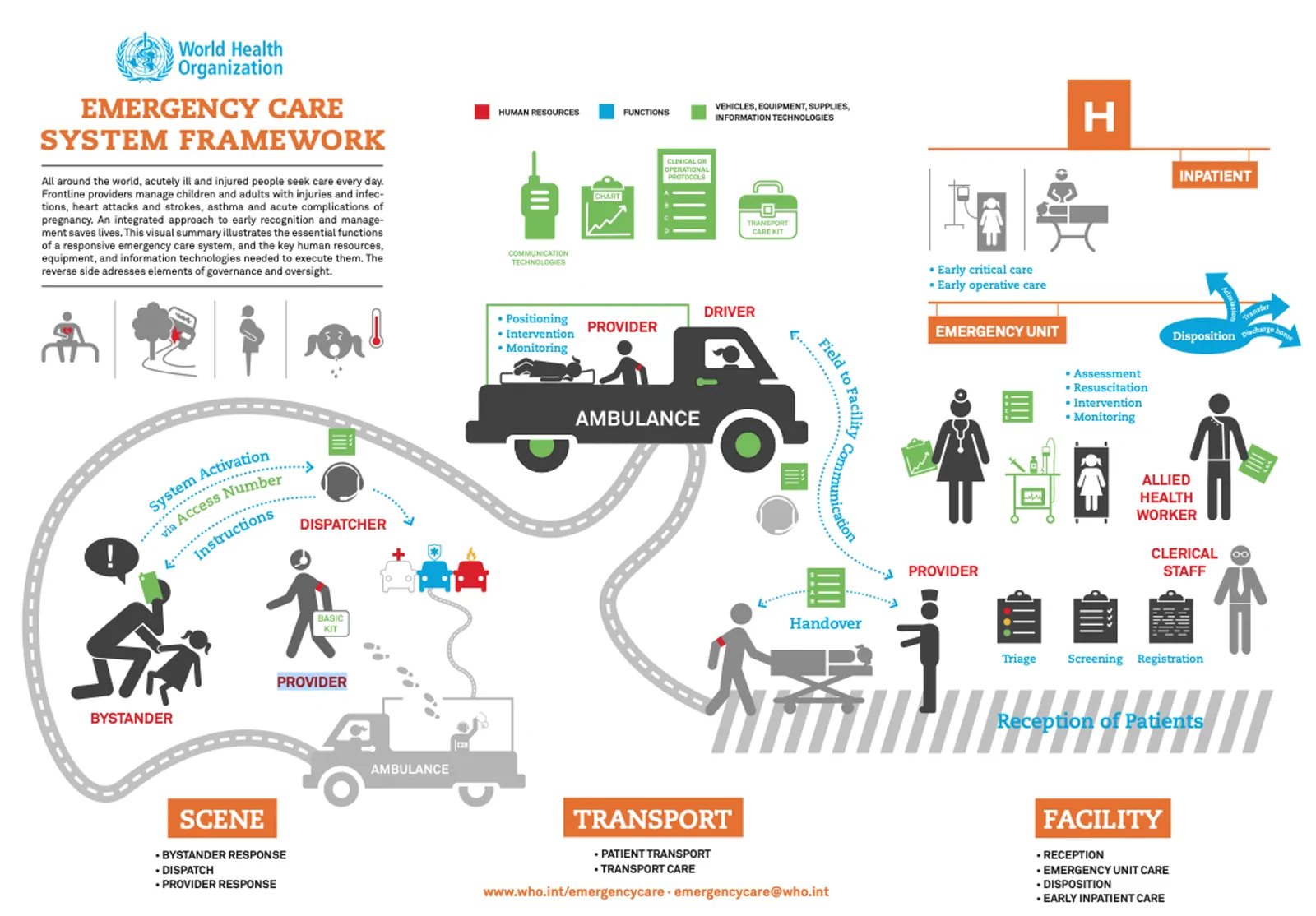
The WHO Emergency Care System Framework WHO: World Health Organization This figure illustrates the WHO Emergency Care System Framework, depicting the continuum of emergency care from the scene of illness or injury through transport and facility-based management. The framework highlights key system components, including bystander response, emergency access and dispatch, prehospital care, patient transport, and emergency unit and inpatient care. Cross-cutting system enablers, such as governance, workforce, equipment, information systems, and communication, are shown as integral to effective coordination and continuity of care. The framework emphasises how timely system activation, coordinated response, and structured handover across care settings are essential to reducing preventable morbidity and mortality. Image credit*: *Adapted from the World Health Organization Emergency Care System Framework [4], used under CC BY-NC-SA 3.0 IGO

Workforce capacity and human resource constraints

Shortage of Trained EMS Professionals

Human resources form the backbone of any effective EMS system, yet Nigeria faces a severe shortage of trained prehospital emergency care professionals [1,29]. Available estimates suggest that fewer than 1,000 formally trained paramedics serve a population exceeding 200 million, a disparity that significantly constrains prehospital emergency care capacity and limits the delivery of timely, life-saving interventions [29,30]. The absence of a nationally standardised EMS training, certification, and accreditation framework further compounds this challenge, as many emergency responders are recruited from other health disciplines without specialised prehospital training [9,26].

This lack of standardisation results in inconsistent competencies, variable quality of care, and limited professional recognition of EMS roles [21,26]. In contrast, EMS systems in high-income countries operate clearly defined workforce hierarchies supported by nationally regulated training curricula, licensing requirements, and continuous professional development frameworks. In the United Kingdom, paramedics are regulated by the Health and Care Professions Council, while structured EMS education and certification in the United States are overseen through nationally recognised scope-of-practice and accreditation standards [27,28,31]. These systems ensure that EMS personnel possess clearly defined competencies and operate within robust clinical governance structures, supporting high-quality emergency care delivery.

Experiences from middle-income countries provide relevant and contextually appropriate lessons for Nigeria. South Africa has developed regulated paramedic training pathways overseen by the Health Professions Council of South Africa, enabling professional recognition and career progression within EMS [32]. Similarly, India has expanded EMS workforce capacity through large-scale public-private partnerships that integrate training, deployment, supervision, and ongoing professional development [33]. These examples demonstrate that structured EMS workforce development is achievable in resource-constrained settings when supported by coherent policy direction, regulatory oversight, and sustained investment.

Emergency Medicine as a Developing Physician Speciality

The limited integration of emergency medicine as a recognised medical speciality further constrains Nigeria’s emergency care system [12,34]. Although emergency medicine training programmes have been introduced in a small number of tertiary institutions, the speciality remains underdeveloped nationally, with only a small cadre of formally trained emergency physicians [34,35]. Consequently, most emergency departments continue to rely on general practitioners or junior medical officers who may lack specialised training in resuscitation, trauma management, and time-critical decision-making.

In health systems where emergency medicine is well-established, emergency physicians play a central leadership role in coordinating multidisciplinary teams, implementing evidence-based protocols, and driving quality improvement initiatives across the emergency care pathway [22,29]. Their presence is associated with improved triage accuracy, more timely interventions, and better patient outcomes, particularly for high-acuity conditions [22,29]. The absence of such clinical leadership in many Nigerian emergency departments contributes to inconsistent care processes, delayed escalation, and sub-optimal outcomes for critically ill and injured patients [12,35].

Expanding postgraduate emergency medicine training programmes, strengthening institutional recognition of the speciality, and integrating emergency physicians into hospital leadership and governance structures will be essential for building sustainable clinical leadership within Nigeria’s emergency care system [29,34]. Without this physician-level capacity, broader EMS and emergency care reforms risk remaining structurally incomplete.

Retention and Migration of the EMS Workforce

High rates of professional migration significantly undermine EMS workforce capacity in Nigeria and other LMICs [18,19]. Poor remuneration, limited career progression opportunities, inadequate working conditions, and insufficient professional recognition drive trained EMS personnel and emergency physicians to seek employment in higher-income settings [18,34]. Global demand for healthcare workers, particularly in emergency medicine, anaesthesia, and critical care, has further accelerated this migration [18,19].

The introduction of Nigeria’s National Policy on Health Workforce Migration represents an important acknowledgement of this challenge at the policy level [17]. However, international experience suggests that migration policies are most effective when coupled with tangible retention strategies, including competitive compensation, safe working environments, access to continuing professional development, and clear career advancement pathways [18,34]. Without meaningful improvements in these areas, Nigeria risks continuing to lose its most skilled emergency care providers, undermining long-term system resilience and emergency preparedness.

Figure 3 illustrates the scale and variability of medical workforce migration across selected countries, highlighting both absolute numbers and proportional losses. In the context of EMS and emergency care systems, such migration represents a critical constraint on workforce availability, leadership capacity, and service sustainability in source countries. Addressing these challenges will require a comprehensive workforce strategy that integrates training expansion, professionalisation, retention incentives, and alignment with broader health system reforms [17,19].

**Figure 3:**
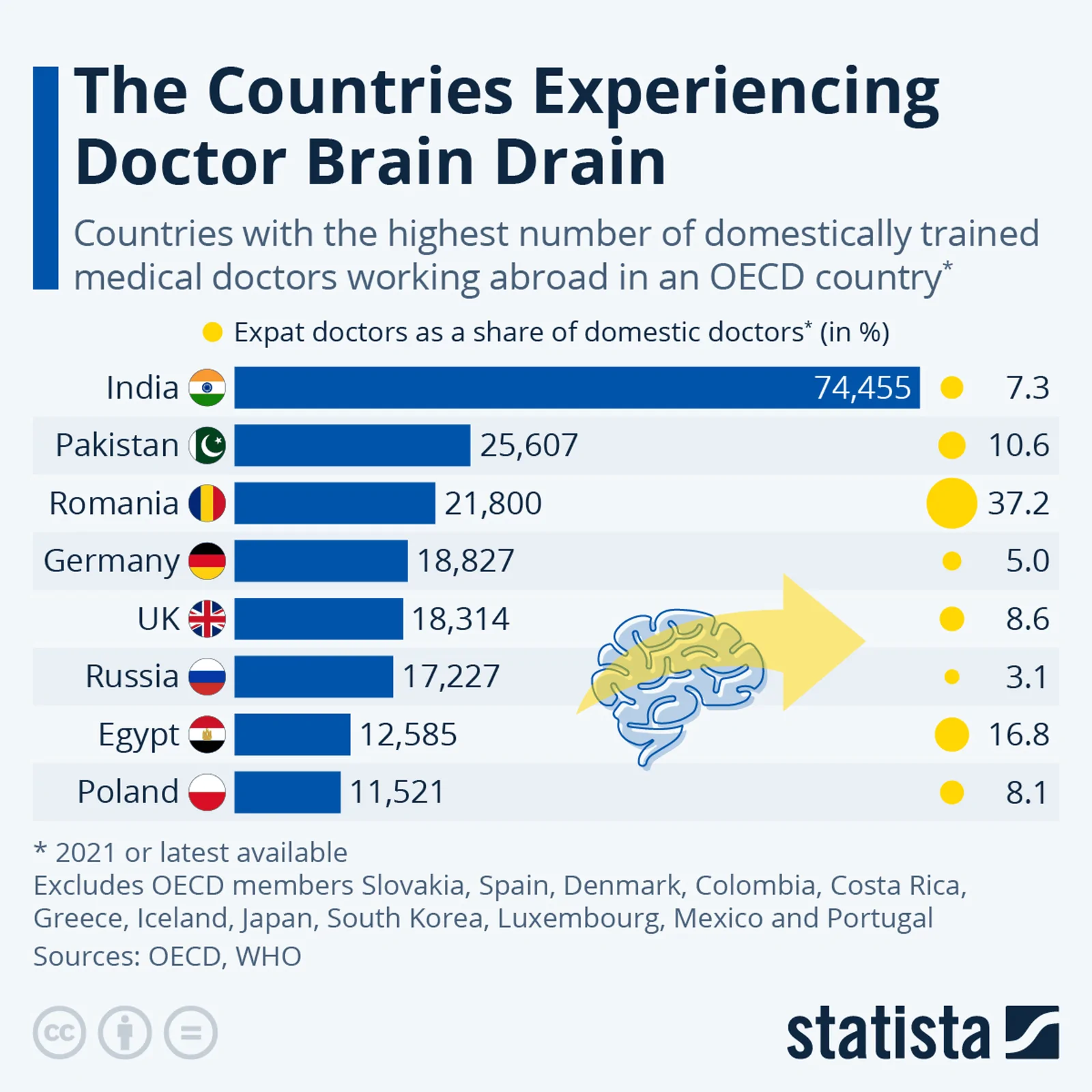
International migration of domestically trained medical doctors from selected countries OECD: Organisation for Economic Co-operation and Development; EMS: Emergency Medical Services This figure illustrates selected countries with the highest absolute numbers and proportions of domestically trained medical doctors practicing abroad in OECD countries. The bars represent the total number of expatriate physicians, while the accompanying markers indicate expatriate doctors as a percentage of the domestic medical workforce. The figure highlights substantial variation in the scale and relative impact of medical workforce migration across countries. In the context of EMS and emergency care systems, such migration represents a critical constraint on workforce availability, leadership capacity, and system sustainability in source countries. Image credit: Adapted from Doctor brain drain: Internationally trained physicians working abroad, by Statista, 2023 [35], under Creative Commons Attribution-NoDerivatives (CC BY-ND) 4.0

Funding and Financial Sustainability of EMS

Inadequate and inconsistent financing remains a fundamental constraint on the development and sustainability of EMS in Nigeria [1,11,31]. Although the NEMSAS was designed to provide publicly funded emergency ambulance services, the programme remains under-resourced, with uneven implementation across states and limited capacity for scale-up [11]. Emergency care financing in Nigeria relies heavily on government budget allocations, donor contributions, and out-of-pocket payments, creating financial instability and constraining long-term planning [20,32].

Nigeria’s public health expenditure remains below international benchmarks, with emergency preparedness and response receiving a relatively small proportion of overall health sector funding [31,32]. This underinvestment limits the expansion of ambulance fleets, procurement of essential equipment, development of communication and dispatch infrastructure, and sustained investment in EMS workforce training and retention [1,21]. In the absence of protected and predictable financing streams, EMS programmes are particularly vulnerable to fiscal volatility and changes in political priorities [23].

The limited integration of EMS into Nigeria’s national health insurance framework further exacerbates financial barriers to access [32]. While recent reforms to the National Health Insurance Authority (NHIA) have expanded coverage for selected health services, comprehensive reimbursement for emergency transport and stabilisation services remains limited [31,32]. As a result, many patients face substantial out-of-pocket costs at the point of emergency care, discouraging timely care-seeking and reinforcing inequities in access, particularly among low-income and rural populations [20,31,32].

Comparative experience from high- and middle-income countries underscores the importance of diversified and protected financing mechanisms for EMS sustainability. In the United Kingdom, emergency care and ambulance services are financed through general taxation and delivered as part of the National Health Service, ensuring universal access irrespective of ability to pay [13,27]. In the United States, EMS financing is supported through a combination of public funding, insurance reimbursement, and local government support, albeit with variability across jurisdictions [14,28]. Middle-income countries such as South Africa and India have adopted hybrid financing models, including public-private partnerships and government-subsidised insurance schemes, to expand EMS coverage while maintaining financial viability [32,33].

From a policy perspective, recognising emergency care as a public good is central to achieving sustainable EMS financing [1,4]. Integrating EMS services, including emergency transport, prehospital care, and early stabilisation, into Nigeria’s national health insurance benefit package represents a critical opportunity to reduce out-of-pocket expenditure and improve equity [31,33]. Complementary strategies, such as earmarked budget allocations for emergency preparedness, performance-based financing, and regulated public-private partnerships, may further strengthen system resilience and accountability [23].

Without sustained and coordinated investment, improvements in governance and workforce capacity are unlikely to translate into meaningful gains in emergency care delivery. Ensuring financial sustainability is therefore not merely a technical financing challenge, but a prerequisite for effective EMS system strengthening and progress toward universal health coverage in Nigeria [1,32].

Discussion

The findings of this narrative review suggest that the limitations of EMS in Nigeria are not isolated operational shortcomings, but manifestations of deeper structural design challenges within the health system. Governance fragmentation, workforce shortages, and financing instability interact in a reinforcing cycle that constrains system performance. As a result, EMS underperformance reflects systemic misalignment rather than the failure of any single policy initiative [16,22].

From a governance perspective, Nigeria’s federal health structure presents a structural tension between decentralised service delivery and the inherently centralised coordination required for effective emergency response. Emergency care depends on standardisation, interoperability, rapid communication, and uniform performance benchmarks, features that are difficult to sustain in the absence of binding national standards and enforceable oversight mechanisms [22,25]. While policy instruments such as the Emergency Treatment and Care Act and the establishment of NEMSAS demonstrate political recognition of emergency care priorities, implementation remains uneven across states [11]. This gap between policy intent and operational reality reflects broader health system governance challenges, including limited regulatory authority, weak accountability mechanisms, and insufficient monitoring infrastructure [23].

Workforce constraints compound these governance limitations. Severe shortages of trained paramedics and the limited institutionalisation of emergency medicine as a recognised speciality restrict both service capacity and clinical leadership [25,29]. Importantly, workforce deficits are not solely attributable to inadequate training pipelines; they are also shaped by professional migration, limited career progression pathways, and weak professionalisation of EMS roles [17-19]. Without a stable and adequately incentivised workforce, governance reforms cannot translate into consistent service delivery improvements. In this sense, workforce instability functions as both a symptom and a driver of system fragility [17].

Financing limitations further entrench these structural weaknesses. Emergency care in Nigeria remains heavily dependent on general budget allocations and out-of-pocket expenditure, with limited integration into national health insurance mechanisms [20,31]. This financing model introduces volatility, constrains long-term planning, and reinforces inequities in access to care [20]. International experience demonstrates that sustainable EMS systems are typically supported by protected and diversified funding streams, reflecting recognition of emergency care as a public good [16,23]. In the Nigerian context, the absence of predictable financing undermines not only service expansion but also workforce retention and governance enforcement capacity [31].

Taken together, these findings indicate that incremental reforms targeting individual system components are unlikely to produce transformative change. Strengthening Nigeria’s EMS requires coordinated, cross-domain policy action that aligns governance authority, workforce professionalisation, and financing mechanisms within a unified national framework [16,23]. Implementation science perspectives suggest that translating existing policy commitments into measurable system improvement will require clearly defined performance indicators, routine evaluation, and sustained political prioritisation of emergency care within broader health sector reforms [23].

Nigeria possesses foundational policy instruments and growing institutional recognition of emergency care as a system priority. The central challenge lies not in policy formulation, but in effective operationalisation, enforcement, and integration [11,23]. Addressing these structural constraints presents an opportunity to strengthen health system resilience, disaster preparedness, and progress toward universal health coverage [16,22].

Limitations

This review has several limitations. As a narrative review, it did not employ a systematic search strategy with predefined inclusion and exclusion criteria and therefore may not have captured all relevant publications. The heterogeneity and limited availability of high-quality empirical data on EMS in Nigeria further constrained the ability to draw quantitative conclusions regarding system performance and outcomes. In addition, reliance on published literature and policy documents introduces potential publication and reporting bias, particularly where government reports may emphasise policy intent over implementation outcomes. Despite these limitations, the narrative approach allowed for a comprehensive system-level synthesis of governance, workforce, and financing challenges, providing a conceptual framework to inform future empirical research and policy evaluation.

Accordingly, the findings of this review should be interpreted as a narrative synthesis intended to inform policy discussion and identify priorities for future research, rather than as a quantitative assessment of the effectiveness of specific EMS interventions.

## Conclusions

Nigeria’s EMS system remains at a critical stage of development, characterized by significant potential alongside persistent structural constraints. This review demonstrates that weaknesses in governance, workforce capacity, and financing are closely interconnected, collectively limiting the effectiveness and equity of emergency care delivery. While recent initiatives, including the establishment of the NEMSAS and broader health sector reforms, represent important progress, their impact has been constrained by fragmented implementation and insufficient system integration.

Strengthening EMS in Nigeria will require unified national leadership, professionalised workforce development, recognition of emergency medicine as a core clinical speciality, and sustainable financing mechanisms that reduce reliance on out-of-pocket expenditure. Importantly, Nigeria’s challenge lies not in the absence of policy frameworks, but in translating existing commitments into coordinated, enforceable, and adequately resourced action. Drawing on lessons from international EMS models, Nigeria has a clear opportunity to strengthen emergency care as a foundational component of health system resilience, disaster preparedness, and progress toward universal health coverage. Future research incorporating primary data from EMS providers, policymakers, and service users will be essential to evaluate reform implementation and guide adaptive strategies for system improvement.
